# Criminal Abortion in Atlanta

**Published:** 1886-05

**Authors:** 


### CRIMINAL ABORTION IN ATLANTA.

The low rumbling thunder is not more certainly presaged by the lightning's flash than the ominous hidden mysteries of criminal abortion, pointed out heretofore in THE JOURNAL, are verified by the proofs of this damnable practice, which have now come to light in the case of Julia Locke. That infanticide should have been followed by the death of a mother while under the care of a practicing physician has given such publicity to the facts of this dark tragedy as to have necessitated a judicial investigation. We find a full confirmation of the stigma resting upon the fair fame of our city in the circumstance that a woman from another State has sought means of ridding herself of the fruit of illicit inter-course by coming here and becoming a victim to the violent measures resorted to for inducing abortion.

The evidence brought out at the coroner's inquest shows that, about the middle of March last, a woman, giving her name as Mrs. Anna Jones, arrived in the city at night and stopped at a hotel. On the following morning she sent a note to Dr. E. H. Green asking him to call, which he did. He engaged board for her at a private house, where he visited her every day. She was taken suddenly ill five or six days after going to this house. One week after she had been there an old colored woman was employed to nurse her. This woman testifies that, on the first night of her stay with the sick woman, Dr. Green called and, after being in the room some time, requested her to go out for water. She informed him there was a supply of water in the room. He requested her to go out and get more, which she did. On her return she found the door locked, and she could not get in. After waiting some time she says the door was opened, and she went in and found Dr. Green had his coat off and was wiping his hands on a towel. There was bloody water in the wash-basin. A few minutes after her admission, Dr. Green put on his coat and picked up a bundle, which was wrapped in a newspaper, and went out, remarking that he would return in a little while.

Dr. Green testified that the woman applied to him to be treated for some uterine trouble, but that he had only prescribed for a severe pain in her head. She claimed to have "missed one month," but "come unwell" while on the train *en route* to Atlanta.

He said he did not suspect her true condition until she had been here one week, and that after he did suspect the trouble abortion had already commenced. He states that she was delivered of a fœtus, he "supposed to be six weeks old," which he wrapped in a newspaper and carried to his office and put into the water sink. The woman grew rapidly worse and died on the second day after the abortion.

*Post-mortem* examination revealed metritis and peritonitis. On the internal surface of the womb, just at the internal os, were found two contusions. Near the fundus was found a piece of placenta.

Quite a number of medical gentlemen were sworn, and with only a single exception, they testified that abortion had been caused by instruments, and death was the result of inflammation produced by them.

The fact that a medical man, who was present at the autopsy, could not or would not see the signs of violence which were patent to those who conducted the examination, does not militate in the least against the correctness and trustworthiness of the observations of these competent members of the medical profession of Atlanta, sustained as they are by the subsequent inspection of other experts who testified to the existence of the uterine lesions indicating the use of instruments.

Although no definite conclusion was reached by the coroner's inquest, a true bill has been found by the grand jury of Fulton county against Dr. E. H. Green, and he was arrested April 7th "on a warrant charging him with assault with attempt to murder," and released under a bond of $1,000.

How far the facts implicate Dr. E. H. Green is now a matter of judicial investigation, and it is not our province to pronounce in advance upon the testimony which may be presented. We are clear, however, in our conviction, from the evidence already before us, that improper measures of violence have been used by some one, inducing abortion and consequent death of the woman, and that a most rigid scrutiny is demanded for verifying upon whom the responsibility rests.

It is high time that this community shall be impressed with the grave reality of the crime of infanticide, and all right-minded men and women must concur in recognizing the destruction of the unborn child as murder, when not authorized by the dire necessity for preserving the life of the mother. Let this case prove a warning to women and to abortionists for all future time.

---

### ITEMS.

FOR the benefit of the New York *Medical Record*, we would say that there is no pol*i*clinic in Vienna, but there is a *poliklinik*.

DR. EDWARD G. JANEWAY has been appointed Professor of Principles and Practice of Medicine in Bellevue Hospital Medical College in place of the late Dr. Austin Flint.

DR. W. F. WESTMORELAND, JR., of this city, is now in New York, where he will remain for several months, attending the surgical clinics at the various hospitals. We will publish in the June number of THE JOURNAL the first of a series of interesting letters from him.

TO THOSE GOING TO ST. LOUIS.—Among the new advertisements in this number of THE JOURNAL will be found the announcement of the "Daisy Line." This line embraces the Western and Atlantic Railroad, Chattanooga and Nashville, and the Louisville and Nashville. It is the shortest line from Atlanta to St. Louis, and offers superior inducements to delegates.

DR. JOHN S. BILLINGS, of Washington, has been chosen to deliver the address in Medicine before the next meeting of the British Medical Association in place of the late Dr. Austin Flint.

A SENSIBLE DOCTOR.—Dr. J. H. Crozier, of Cedar Springs, Ga., in a letter of recent date, says: "I appreciate your JOURNAL very highly; think it is getting better every issue. Like it better than any Southern journal. I can't practice without it."

J. H. BATES, the well-known advertising agent, 41 Park Row, New York, has purchased of S. M. Pettengill, the Newspaper Advertising Agency, conducted by him in New York city so successfully for almost forty years, and has merged it with his own, thus increasing the stability and importance of a house already appreciated by the business public.

AS AN illustration of the wide circle of readers which is reached by the articles published in THE ATLANTA MEDICAL AND SURGICAL JOURNAL, we would state that our mail brings us a postal card from Dr. Gust. Beck, of Berne, Switzerland, editor of the *Zeitschrif fur Arztliche Polytechnik*, asking us for a copy of Dr. Virgil O. Hardon's article on "The Use and Abuse of Pessaries," which was published in our December issue. We take pleasure in acknowledging this compliment to THE JOURNAL, as well as to the author of the article in question.

"THE Other Side of Cocaine—the Bad Side" was the subject of a paper presented by Dr. A. W. Calhoun, of this city, to the State Medical Association in Augusta last week. The doctor argues strongly against the use of cocaine in cataract operations by extraction. He stated that before he began the use of cocaine from 95 to 97 per cent. of all cataract operations by extraction proved successful, and that in a large number in which cocaine had been used, he had bad results. He has abandoned its use altogether in operations by extraction.

DR. T. GAILLARD THOMAS has removed from 294 Fifth Avenue, New York, to 600 Madison Avenue, between Fifty-seventh and Fifty-eighth streets.

THE following letter was received by a physician in this city a few days since :

——— INDIANA.

Mr ——— M D
Dear Sir ie am A homapanic student of Anatomy of the human Body and deases of Woman
if you have some goots book of that kind please send Mee pricelist of some of them how is the oald Castal
Direct —— Indiana, Mr. —— ———
yours truly
Mr. ——— M. D.

THE GENU-PECTORAL POSITION.—We have received the advance sheets of an article by Dr. H. F. Campbell, of Augusta, Ga., upon "The Genu-Pectoral Position—Its Value in Impeded Uterine Reduction and in the Prolonged Nausea and Vomiting of Pregnancy." This paper was read at the last meeting of the American Gynæcological Society, and has for its object "the discussion of some strictures and misconceptions in regard to it which have appeared" since his first paper upon this subject was published in 1876. The author successfully defends his claim as the originator of the genu-pectoral position in gynæcology as distinguished from Bozeman's knee-elbow position. That such a defense is not uncalled for is illustrated by the fact that in a recent article by Betrix, in the *Nouvelles Archives d' Obstetrique et de Gynecologie* for January, 1886, p. 35, we find this position described as "that of Bozeman or the genu-pectoral." But our observation inclines us to the opinion that upon this side of the Atlantic there is very little such misapprehension of the facts and very little disposition to deprive the eminent Georgia gynæcologist of the honor which is justly due him.

A HYSTERICAL CRIMINAL.—A dead infant was sent by post to a French priest. The police made inquiries, and arrested a young servant girl, who confessed her guilt. She immediately afterwards completely lost the power of speech, which has not returned. During her trial, she made known her answers by signs and writing. She has been examined by Dr. Brouardel, who pronounces her to be hysterical. She is aphasic and anæsthetic; pins stuck into different parts of her flesh produce neither pain nor bleeding. Dr. Brouardel does not suppose that her dumbness is assumed; but adds, as it is a symptom of hysteria, and she is hysterical, it is impossible to be certain. The patient, who had previously committed other crimes, has been sentenced to three years' imprisonment.—*British Medical Journal, April 10th.*

SHALL THERE BE LIBERTY OF DISCUSSION IN MEDICAL MEETINGS?—A case in which all physicians are interested is now under trial in the courts of Philadelphia. The facts, as explained by the Philadelphia *Medical Times*, are briefly these: Dr. Carl Seiler, several months ago, before the Alumni Association of the College of Pharmacy, of this city, and within the hall of the college, delivered a lecture upon "Hay-Fever." After he had explained the modern views of the neurotic nature of the disease, he stated the fact that it was often excited by pollen from various plants, as well as by particles of dust in the air; and he pointed out the fact that in the treatment, all powders are therefore injurious, since they act as irritants to the inflamed mucous membrane. In impressing this point upon his audience, speaking extemporaneously, he condemned all powders for this reason, and told his audience not to use them. Among the examples which he mentioned was a proprietary article in the form of a snuff, which had been advertised in Philadelphia as a "cure" for hay-fever. The professional experience of the lecturer, and his knowledge of the nature of the disease in question, he believed, enabled him to form, and qualified him to express, a positive opinion as to the dangers of a remedy of the kind indicated when used in this highly objectionable manner. For this statement, made before a medical audience (for pharmacy is a branch of medicine), Dr. Seiler has been prosecuted by the owner of the nostrum, who claims exemplary damages for injury to his business. If the case is decided in favor of the plaintiff in this case, medical men, as our contemporary remarks, may consider it as an intimation that the patent-medicine business has grown so powerful that it may now issue its mandates directly to the profession, and that hereafter, if proprietary medicines are mentioned at all in our discussions, it shall only be in terms of commendation and with due respect.—*Boston Medical and Surgical Journal, April 15.*

A SAD DEATH.—The death of Mrs. Dr. N. O. Harris will bring sadness to the hearts of her many friends. Her death occurred at her home in this city, April 23d. We extend to the bereaved husband our heartfelt sympathy. At a called meeting of the Atlanta Society of Medicine, held April 26, the following resolutions were adopted:

WHEREAS, our esteemed professional brother, Dr. N. O. Harris, has recently met with the loss by death of a loving and devoted wife: therefore, be it

*Resolved*, That we, the members of the Atlanta Society of Medicine, of which he is an active and honored officer, do hereby tender to him, in this his hour of affliction, our sincere sympathy and condolence and the assurance that our hearts beat as one in grief for the irreparable loss which he has sustained.

That these resolutions be sent to Dr. Harris and published in the medical journals and daily papers of this city.

W. S. ELKIN,
ARCH. AVARY,
VIRGIL O. HARDON,
Committee.

GELATIN-COATED GRANULES OF RARE DRUGS AND ALKALOIDS.—We think no physician will to-day deny the great advantages to medicine resulting from the work done by manufacturing pharmacists during the last decade. This gain to medicine is especially noticeable when we review the work done by those pharmacists who have been working in harmony with the interests of the medical profession. With the advantages of capital and skilled investigators and experimenters, such pharmacists have been able to place before physicians far more concentrated, accurate, convenient and agreeable methods of medication. Among these improved forms for administering medicines may be mentioned the granule, which is especially serviceable in administering medicines, the dose of which is small, such as the rare drugs and alkaloids which recent therapeutic research has added to the materia medica. No house has been more active and successful in its efforts to establish the practice of pharmacy and medicine on a scientific basis than that of Messrs. Parke, Davis & Co., who have not only been identified with the introduction of many new and valuable drugs, but are always endeavoring to improve upon and perfect the methods of medication in vogue.

METEOROLOGICAL SUMMARY FOR MARCH, 1886, STATION, ATLANTA, GA.

| | |
|---|---|
| Mean Temperature, | 50.1 |
| Highest Temperature, March 20th, | 73.0 |
| Lowest Temperature, March 11th, | 27.0 |
| Monthly Range of Temperature, | 46.0 |
| Greatest Daily Range of Temperature, | 27.5 |
| Least Daily Range of Temperature, | 5.5 |
| Mean Daily Range of Temperature, | 16.6 |
| Prevailing Direction of Wind, | N. W. |
| Total Movement of Wind, | 8,535 Miles. |
| Highest Velocity of Wind and Direction, | 33 Miles, W. |
| Total Precipitation. | 11.16 |
| No. of Days on which .01 inch or more of Rain or Snow fell, | 11 |

